# Fabrication of Hierarchical Indium Vanadate Materials for Supercapacitor Application

**DOI:** 10.1002/gch2.202000002

**Published:** 2020-09-28

**Authors:** Balachandran Subramanian, Manimuthu Veerappan, Karthikeyan Rajan, Zheming Chen, Chengzhi Hu, Fei Wang, Feng Wang, Mingshu Yang

**Affiliations:** ^1^ Beijing National Laboratory for Molecular Sciences Key Laboratory of Engineering Plastics Institute of Chemistry Chinese Academy of Sciences Zhongguancun North First Street 2 Beijing 100190 P. R. China; ^2^ Department of Mechanical and Energy Engineering Southern University of Science and Technology Nanshan District Shenzhen Guangdong 518055 P. R. China; ^3^ Department of Electrical and Electronic Engineering Southern University of Science and Technology Nanshan District Shenzhen Guangdong 518055 P. R. China; ^4^ Engineering Research Center for Hydrogen Energy Materials and Devices College of Rare Earths (CORE) Jiangxi University of Science and Technology Ganzhou Jiangxi 341000 P. R. China

**Keywords:** InVO_4_, pseudocapacitors, silica‐encapsulated InVO_4_

## Abstract

Transition metal orthovanadates are emerging 2D materials for promising electrochemical energy storage applications. Facile hydrothermal method for nanocrystalline indium vanadate (InVO_4_) semiconducting materials’ fabrication is economical because of its direct chemical synthesis. X‐ray diffraction studies, field emission scanning electron microscope (SEM) images, transmission electron microscopy (TEM), and photoelectron X‐ray spectrum are used to describe the semiconductor materials as synthesized. InVO_4_ microspheres have attracted a lot of attention in the energy and environmental sector. These microsphere‐derived semiconductor materials are recognized to offer the advantages of their large surface area, tunable pore sizes, enhanced light absorption, efficient carrier (electron–hole) separation, superior electronic and optical behavior, and high durability. From the results of SEM and TEM, InVO_4_ shows a microsphere construction with a mixture of nanosized particles. Diffuse reflectance UV–visible measurements are used to determine the bandgap, and it is found to be 2.1 eV for InVO_4_. The electrochemical analysis reveals a superior performance of the pseudocapacitor with hydrothermally derived microspheres of InVO_4_. Alongside an improved pseudocapacity, developed after 4000 cycles, it has excellent cycling stability with a retention of ≈94% of its original specific capacitance efficiency.

## Graphical abstract

A simple hydrothermal method is utilized to synthesize InVO_4_ and InVO_4_–SiO_2_ nanostructures. InVO_4_ exhibits typical hierarchical morphology with a superior electrochemical performance of 1710 F g^−1^ at 2 A g^−1^. Also, encapsulation of SiO_2_ on InVO_4_ provides an added potential in energy storage applications. A stability retention of ≈91% pseudocapacitance is achieved after 4000 cycles for InVO_4_.

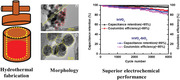

## 1 Introduction

In recent times, scientists have been paying more attention in the significance of microarchitectures. 3D hierarchical architectures are built from the nanostructured building blocks of nanoparticles, nanoplates, nanoribbons, and nanorods. Hierarchical nanostructures are essential foundations of a large number of current and upcoming technologies, including chemical and biological molecules delivery;^[^ ^1^ ^]^ structural self‐healing materials;^[^ ^2^ ^]^ plasmonic;^[^ ^3^ ^]^ photosynthetic assemblies;^[^ ^4^ ^]^ separations, sensing, and catalysis;^[^ ^5^ ^]^ fabrication of photonic;^[^ ^6^ ^]^ and constructions of electronic displays.^[^ ^7^ ^]^ These materials are essential components and building principles for the evolution to propose a more consistent, more efficient, and eco‐friendly multidisciplinary approach. The microsphere's external and internal structures are essential to their function and performance. Since last decade, material chemists have established that the complex hierarchical structured materials can be fabricated with high control and efficiently using various chemical strategies.^[^ ^8^, ^9^, ^10^ ^]^ Such significant architectures have the features of micrometer‐ and nanometer‐scaled construction blocks, which behave differently from those of their morphological structures.^[^ ^11^, ^12^ ^]^

Indium vanadate (InVO_4_) belongs to the family of orthovanadate oxide with a narrow bandgap (2.0 eV). It was found to be a significant fundamental semiconductor material because of its potential applications.^[^ ^13^ ^]^ The exclusive optical and electrical properties are utilized in different areas of research, including degrading toxic organic pollutants,^[^ ^14^, ^15^ ^]^ air purification,^[^ ^16^ ^]^ water splitting,^[^ ^17^, ^18^ ^]^ and lithium electrode.^[^ ^19^ ^]^ InVO_4_ has excellent photocorrosion stability, and its adequate absorption in the visible light is because of the specific crystal structure. The visible light activities of InVO_4_ were recognized to sub‐bandgap transitions from impurity states. Still, the impurities also help the superfluous recombination, even if they are beneficial for the extended absorption in the visible range.^[^ ^20^, ^21^, ^22^ ^]^ There are two polymorphic InVO_4_ forms: the monoclinic and the orthorhombic phase. The crystal phase of InVO_4_ has comprised of VO_4_ tetrahedral edges shared by InO_6_ groups that create monoclinic chains and In_4_O_6_ small groups in the orthorhombic phase.

Nowadays, various methods have been found to enhance the semiconductors’ activity of metal nanocatalysts. The sacrificial self‐stabilization of metal nanomaterials is carried out through encapsulation of nanocatalysts with semiconductor metal oxide.^[^ ^23^, ^24^, ^25^, ^26^, ^27^, ^28^, ^29^, ^30^ ^]^ Core–shell arrangement was frequently employed for stabilizing the heterogeneous nanomaterial catalysts since their unique construction can avoid the aggregation of nanosized catalysts in aqueous solution and prevent the nanocatalysts from sintering at high temperatures.^[^ ^31^, ^32^ ^]^ Besides the surface chemical interaction between core (nanocatalysts) and shell (supports), it may also develop the semiconductor metal oxide electron‐transfer activity.^[^ ^33^ ^]^ Silica had been extensively used as a “stabilizer” of metal nanocatalysts because of its high thermal stability and tolerance toward acidic media.^[^ ^34^, ^35^ ^]^ In the past decade, mesoporous silica nanoparticles have gained significant attention for multiple applications due to their desirable features such as physicochemical stability, tunable microstructure, and straightforward surface functionalization.

Higher performance, extended cycle stability, and low maintenance cost found supercapacitors as a promising candidate for energy storage. Also, supercapacitors have higher power density, longer life cycles than batteries, and much higher energy density than a conventional dielectric capacitor. Pseudocapacitors have a primary scientific and technological importance, which makes them very promising for hybrid vehicle applications and portable electronic devices that require high electrical power. The working principle of a pseudocapacitor relies on a fast, reversible, redox reaction of the active electrode material where outstanding capacitive ability is essential. In this view, a facile hydrothermal method was used to synthesize InVO_4_ hierarchical microstructures and InVO_4_–SiO_2_ nanosheets.

## 2 Results and Discussion

**Figure 1:**
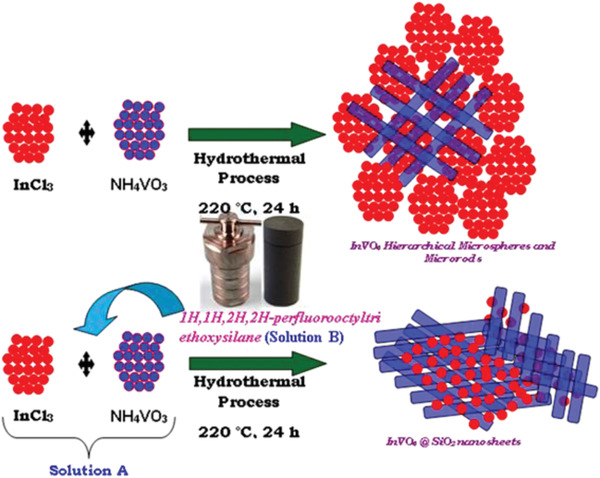
Schematic representation of InVO_4_ hierarchical microspheres and microrods preparation and InVO_4–_SiO_2_ nanosheets.

**Figure 2:**
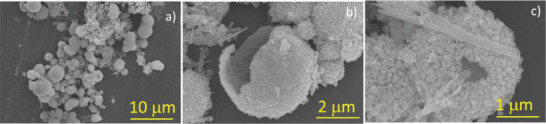
FESEM images of InVO_4_ at different magnifications: a) 10 µm, b) 2 µm, and c) 1 µm.

**Figure** **1** describes the schematic representation of the growth process by the hydrothermal method and the corresponding microstructures’ evolution of InVO_4_ and InVO_4_–SiO_2_. The results of field emission scanning electron microscope (FESEM) showed that the hydrothermal method provided an essential route to InVO_4_ morphology control. The structure and morphological integrity of the InVO_4_ materials are well maintained. In **Figure** **2**a,c, the spherical‐like morphology can be seen. Also, the small number of microrods positioned on the surface of the microsphere is observed. The broken hierarchical microsphere clearly described the building blocks of nanostructures to microsphere (Figure 2b). According to the sample preparation and calcination process, the particles from nanosize to microsize tend to accumulate. A large number of aggregates organize themselves into hierarchical microspheres and microrods of definite structure and inhomogeneous size when using high temperature with a reaction time of 24 h (see Figure 2). Scanning electron microscope (SEM) images show that an average diameter of InVO_4_ microspheres is between 1 and 2.5 µm. Moreover, these microspheres comprised several uniform sized nanoparticles, as shown in Figure 2b,c. The broken hollow microspheres seen clearly confirm the formation process of hierarchical structures. With SiO_2_ addition, InVO_4_–SiO_2_ composites’ morphology resulted with microsheets’ architectures is attached with the several independent sheet‐like structures to form an irregular morphology (Figure S3, Supporting Information). The microsheets were self‐aggregated and created a large rock‐like structure.

**Figure 3:**
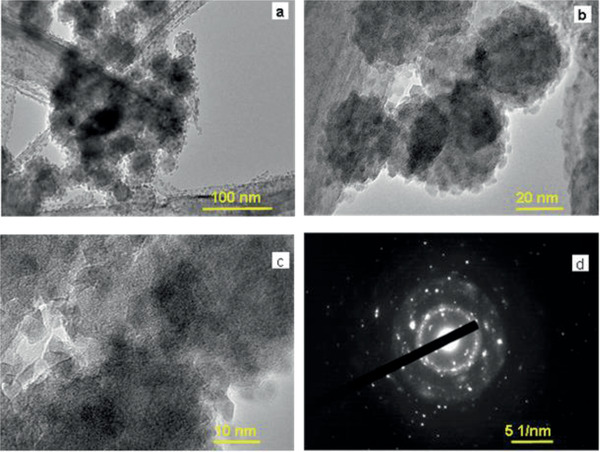
High resolution transmission electron microscope images of InVO_4_ at different magnifications: a) 120 K, b) 400 K, c) 800 K, and d) SAED pattern.

**Figure 4:**
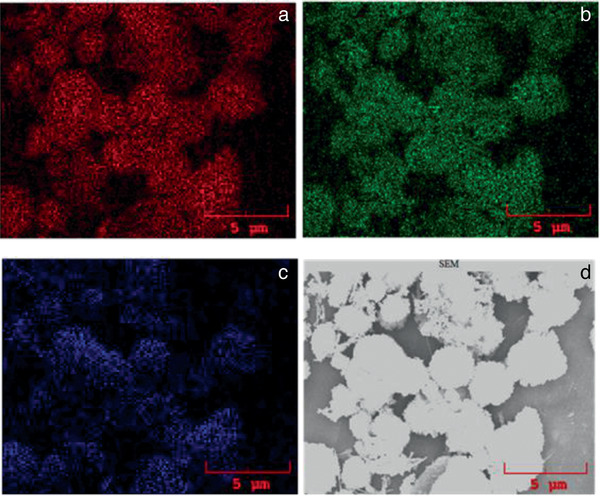
Elemental color mapping InVO_4_ from FESEM analysis: a) In, b) V, c) O, and d) corresponding SEM images.

Transmission electron microscopy (TEM) analysis was performed to investigate the microstructure and crystal growth orientations. **Figure** **3**a reveals the spherical morphology of an InVO_4_ microsphere, which is composed of tiny nanoparticles aggregated to form a hierarchical microsphere. The nanoparticles have 3D octagonal and hexagonal shapes, and they are attached with one another (Figure 3c). Several pores are seen from the boundary of the microsphere in Figure S4a (white dots, Supporting Information). InVO_4_ and InVO_4_–SiO_2_ both are highly crystalline, which are seen throughout the particle. In InVO_4_–SiO_2,_ the average size of the microsheet was found to be 3–6 µm, and a fragile layer of SiO_2_ is present on the surface of microsheets. The smooth surfaces of the InVO_4_ microsheets and microspheres became rougher after coated with an additional layer of SiO_2_. In–L, V–K, and O–K elemental color maps obtained from FESEM analysis have same textural behavior (**Figure** **4**). Therefore, elemental mapping offers a significant proof that In, V, and O are present in the purest form on the synthesized product.

**Figure 5:**
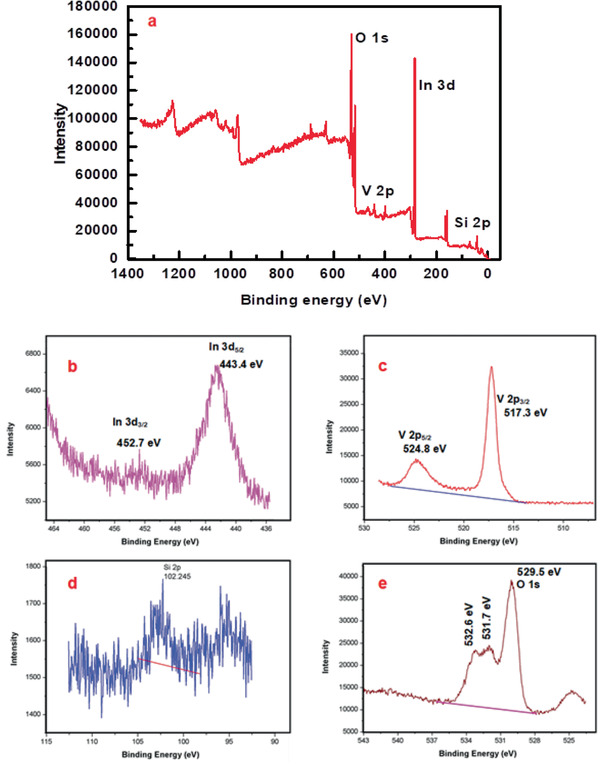
XPS spectra InVO_4_: a) survey spectra, b) In 3d, c) V 2p, d) Si 2p, and e) O 1s.

The X‐ray photoelectron spectrometer (XPS) technique was carried out to analyze the specific surface composition and bonding nature of the samples. The In 3d spectrum (**Figure** **5**b) is depicted to be In 3d_5/2_ and In 3d_3/2_ doublets, which are in good agreement with the binding energy levels of the In^3+^ oxidation state peak positions that have centered at 443.8 and 452.7 eV, respectively. Vanadium V 2p spectrum in Figure 4c suggests that V^5+^ oxidation state has a doublet peak (517.2 eV for V 2p_3/2_ and 524.5 eV for V 2p_1/2_). Two peaks located at 529.5 and 531.3 eV belong to O 1s spectra for InVO_4_ or InVO_4_–SiO_2_. These peaks are associated with surface lattice oxygen and adsorbed (O_ads_) oxygen species, respectively (Figure 5e).^[^ ^36^, ^37^, ^38^ ^]^ Higher levels of O_ads_ (O^−^, O^2−^, or O_2_^2−^) are considered to have mean higher vacancy densities in oxygen, which play a significant role in the electron transfer for photocatalytic applications.^[^ ^39^ ^]^

**Figure 6:**
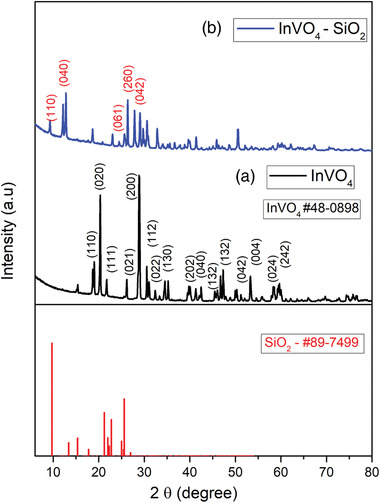
XRD patterns of a) InVO_4_ and b) InVO_4_–SiO_2_ matched with the corresponding SiO_2_ JCPDS reference.

The X‐ray diffraction (XRD) patterns of the InVO_4_ and InVO_4_–SiO_2_ materials are displayed in **Figure** **6**. The diffraction peaks of the orthorhombic InVO_4_ can be indexed to (110), (020), (111), (021), (200), (112), (022), (130), (202), (040), (132), (042), (004), (024), and (242) planes with the corresponding 2θ positions of 18.59°, 20.84°, 23.02°, 24.86°, 27.09°, 31.07°, 33.05°, 34.42°, 35.20°, 41.67°, 42.40°, 44.98°, 55.85°, 60.27°, and 60.96°, respectively. The standard reference pattern indexed to InVO_4_ is JCPDS #48–0898 and it belongs to orthorhombic crystal structure.^[^ ^40^ ^]^ After the encapsulation with SiO_2_, the peak intensities of the InVO_4_–SiO_2_ XRD pattern were slightly decreased because of the amorphous nature of silica. The indexed diffraction planes in Figure 6b are (110), (040), (061), (260), and (042) which correspond to the SiO_2_ (JCPDS 89–7499). Small amounts of trace element V_2_O_5_ were observed for the low‐intensity peaks in both InVO_4_ and InVO_4_–SiO_2_ XRD patterns. However, the morphological analysis from energy dispersive X‐ray spectroscopy mapping, TEM, and FESEM images confirm that the majority of the material is related to InVO_4_ and InVO_4_–SiO_2_.

**Figure 7:**
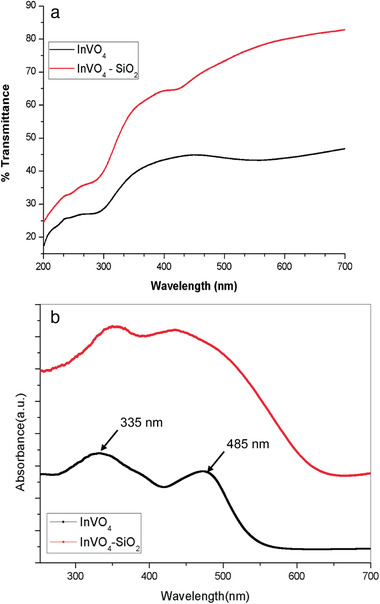
a) UV–vis spectra of InVO_4_ and InVO_4_–SiO_2_ (in solution). b) UV–vis DRS spectra of InVO_4_ and InVO_4_–SiO_2_ (in solid).

**Figure** **7**a displays the InVO_4_ and InVO_4_–SiO_2_ optical absorption behavior obtained from UV–visible spectroscopy. InVO_4_–SiO_2_ exhibits superior visible light absorption properties compared to InVO_4_. The prepared InVO_4_ showed that the absorption wavelength ranges from ultraviolet to visible light region, and the absorption edge is found to be at ≈485 nm. InVO_4_ microsphere revealed a clear redshift and prominent absorption improvement compared to silica‐encapsulated InVO_4_. The noticeable light absorption of indium vanadate is close to InVO_4_–SiO_2_, suggesting that the InVO_4_ microsphere can be useful for visible light reactions. Optical absorption property near the absorption edge follows the equation^[^ ^41^, ^42^, ^43^ ^]^

(1) $$\begin{array}{c} \alpha h\nu =A{\left(h\upsilon -E_{\text{g}}\right)}^{n/2} \end{array}$$

It was found to be ≈467 nm, which is equal to 2.6 eV (InVO_4_–SiO_2_) energy bandgap (*E*_g_). In comparison, the InVO_4_ microsphere absorption edge shifted to a higher wavelength (518 nm) with an average bandgap of 2.2 eV.

Furthermore, the band edge place of the conduction band (CB) as well as valence band (VB) of InVO_4_ and InVO_4_–SiO_2_ can be found making use of the following empirical equation according by Moleken electronegativity theory

(2) $$\begin{array}{c} E_{\text{CB}}\;=X-E_{\text{C}}-E_{\text{g}}\text{/}2 \end{array}$$

(3) $$\begin{array}{c} E_{\text{VB}}=E_{\text{CB}}+E_{\text{g}} \end{array}$$

where *E*_VB_ and *E*_CB_ are the conduction band and valence band edge capacities of InVO_4_ or InVO_4_–SiO_2_; specifically, *E*_g_ is the bandgap energy of the materials, *E*_C_ is the energy of free electrons on the hydrogen scale (normally 4.5 V), and *X* is the electronegativity of the semiconductor. Here, the *X* value of InVO_4_ is found to be 5.03 eV specifically.^[^ ^44^, ^45^ ^]^ From the above equations, their corresponding CB and VB values of InVO_4_ were −0.57 and 1.63 eV, respectively.

Photoluminescence (PL) analysis was performed to understand the photocatalytic mechanism. It is understood that all the peak intensity is related to high or low electron–hole separation rate. PL spectra of InVO_4_ microsphere (Figure S6, Supporting Information) show that InVO_4_ could suppress electron–hole pairs recombination, and more numbers of electrons and holes take part toward the capacitive efficiency in supercapacitor applications.

**Figure 8:**
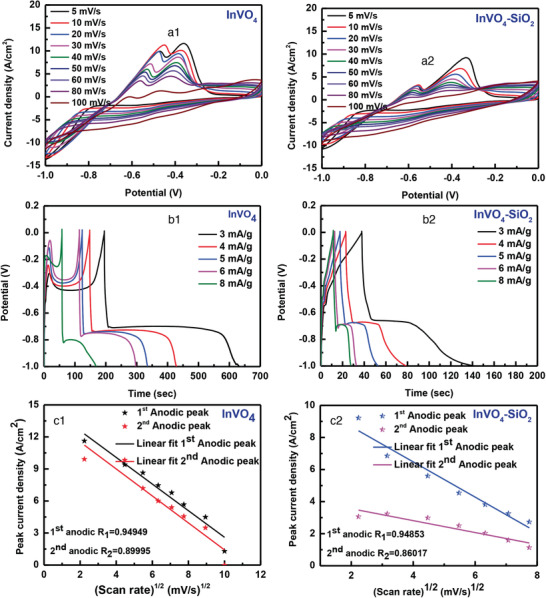
a1) CV curves of InVO_4_, a2) InVO_4_–SiO_2_ at 2 mV CV curves of InVO_4_ at different potentials (5–100 mV). b1,b2) Galvanostatic charge–discharge curves of the InVO_4._ Charge–discharge behaviors of the electrodes potential windows from −1.0 to −0.4 V at 3 to 8 A g^−1^. c1,c2) Anodic peak current versus square root of scan rate for InVO_4_ and InVO_4_–SiO_2_.

Greater pseudocapacitors would exhibit characteristics including high utilization functionality with the subsequent mass loading of electroactive materials. We have tested the InVO_4_ and InVO_4_–SiO_2_ hierarchical structures as assigned electrodes in a three‐electrode configuration system with aqueous electrolytes via cycle voltammetry and galvanostatic charge–discharge (GCD) measurements with 1 m KOH electrolyte. In **Figure** **8**a1,a2, typical cyclic voltammograms (CV) of InVO_4_ and InVO_4_–SiO_2_ were in the potential range from −1.0 to 0.2 V with various scan rates such as 5, 10, 20, 30, 40, 50, 60, 80, and 100 mV s^−1^. It is evidently detected that the characteristic anodic as well as cathodic peaks are validated electrochemically active and are directed by Faradaic redox reaction. Figure 8a1,a2 presents the direct symmetry of scan rates and peak currents assigning the direct diffusion of hydroxyl radical anions (OH^−^) at the responsive active site of the electrode surface area. The well‐defined redox peak of M—O/M—O— or OH— (M = In and V ions) is linked to reversible Faradaic reactions (Faradaic mechanism), which can be observed with all the electrode materials, indicating their pseudocapacitive properties. InVO_4_ microsphere revealed a pair of redox peaks at −0.36 and −0.47 V, while for InVO_4_–SiO_2_ microsheets, redox couple at −0.40 and 0.57 V. The InVO_4_ and InVO_4_–SiO_2_ microsheets showed the identical CV form associated with the well‐defined peaks of the compound, which showed the electrochemical process by one anodic peak at 0.392 V and two small cathode peaks at 0.198 and 0.188 V, respectively. The shape of the CV curves depicts two redox peaks during a cathodic and an anodic sweep different from the electric double layer capacitance defined by rectangular CV curves. These shapes were related to a typical pattern of pseudocapacitive material, i.e., increase in scan rate results in the peak shift toward negative potential, which reflects the internal resistance of the semiconducting material.^[^ ^46^ ^]^ However, the integrated zone of the InVO_4_ electrode current–potential curves was more significant than the InVO_4_–SiO_2_. It indicates a higher capacity that might be extracted from a nanoarchitecture. The InVO_4_ microsphere is induced by Faradaic redox reactions, with an increase in the sweep rate, which increases the anodic peak current density and decreases the cathode peak current density. It implies to low electrode resistance and fast redox reactions at the InVO_4_ and KOH electrolyte solutions’ interface.^[^ ^47^, ^48^, ^49^, ^50^ ^]^

Besides CV, the GCD measurements were performed in a potential window of 0–0.5 V (vs saturated calomel electrode). The GCD test curves show that InVO_4_ has excellent capacity activity and higher electrochemical reversibility. InVO_4_ curves with different current densities of 3–8 mA are shown in Figure 8b1,b2. GCD curves are linear and symmetrical, showing excellent reversibility and material charging–discharging properties. For increasing current densities, the duration of the charging–discharge process decreases slowly. Also, the sudden drop within a few seconds at the discharge section indicates the internal resistance of InVO_4_. Even after the inclusion of SiO_2_, the internal resistance of the material is still carried forward in InVO_4_–SiO_2_ material. However, InVO_4_ indicates the highest capacitance than InVO_4_–SiO_2_ for the more extended charge–discharge period. Their specific capacities are calculated using Equation (1) from Figure 8b1,b2

(4) $$\begin{array}{c} C_{\text{m}}=\frac{I\Delta t}{m\Delta V}(\text{F}\;{\text{g}}^{-1}) \end{array}$$

where *C* (F g^−1^) is the specific capacitance, *A* is the discharge current, Δ*t* (s) is the total discharge time, *m* (g) is the active materials (InVO_4_ and InVO_4_–SiO_2_), and Δ*V* (V) is the potential range during discharge. Based on the above equation, the calculated results depicted in Figure 8b1 that the InVO_4_ material has a specific capacity of 1710 F g^−1^ with a current density of 5 A g^−1^. The excellent performance of supercapacitor electrodes must behave with superior rate performance. Typically, precise reduction in capacitance descends with the increased discharge current density. At low current densities, the ions (electrolytes) have the access to all open pores of the electrode, which are capable of penetrating the InVO_4_ internal structures. At higher current densities, the active use of electrode materials is only limited to the outer surface.

GCD curves show excellent high rate efficiency, efficient charge‐transfer interlayer transmission, and ideal sample capacitive properties. Two of the super capacitive charge–discharge mechanisms were suggested as follows: i) on electrolyte (H^+^, Li^+^, Na^+^, K^+^) cations’ surface adsorption/desorption in InVO_4_, and ii) it includes electrolyte cation intercalation/deintercalation in the bulk of InVO_4_ microsphere^[^ ^47^ ^]^

(5) $$\begin{array}{c} {\text{InVO}}_{4}+\text{electrolytecations}\left({\text{K}}^{+}\right)+{\text{e}}^{-}\;↔\;{\left(\text{InVOO}\;{\text{K}}^{+}\right)}_{\text{surface}} \end{array}$$

(6) $$\begin{array}{c} {\text{InVO}}_{4}+\text{electrolytecations}\left({\text{K}}^{+}\right)+{\text{e}}^{-}\;↔\;\text{InVOO}\;{\text{K}}^{+} \end{array}$$

The linear connection observed in between the redox peak current and the square root of scan rate (Randles–Sevcik relationship) recommends that the electrode surface reactions for different scan rates were diffusion‐controlled processes (Figure 8c1,c2).^[^ ^51^, ^52^ ^]^ The observed redox peaks implied OH^−^ diffusion from the electrolyte (KOH) to the electrode surface (InVO_4_ or InVO_4_–SiO_2_) during reduction and from electrode to the electrolyte during the oxidation procedures.

**Figure 9:**
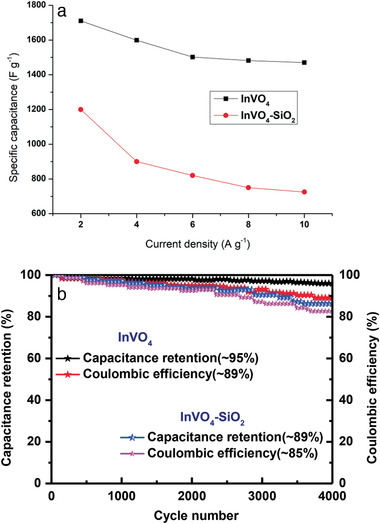
a) Galvanostatic charge–discharge curves of the InVO_4_ and InVO_4_–SiO_2_. Charge−discharge behavior of the electrodes potential windows from −1.0 to −0.4 V at 2–10 A g^−1^. b) The specific capacitances retention and Coulombic efficiencies for 4000 cycles at 5.0 A g^−1^.

The cycle life of the supercapacitor is another crucial factor in determining its efficiency. Long‐term cycling stability is critical for the use of InVO_4_ electrode material for supercapacitors in real time. As shown in **Figure** **9**b, the InVO_4_ microsphere electrode cycling stability was calculated by repeating the charge/discharge test at a high current density of 5 A g^−1^ for 4000 cycles. The InVO_4_ electrode capacitance efficiency increases gradually during the 500th cycle between 1655 and 1710 F g^−1^. At the initial cycles, the real potential increases slowly, and later there is a slight reduction observed. This phenomenon infers pseudocapacity at the initial activation of Faradaic electrode material.

Further, the capacitance has almost no loss following 2000 cycles. This shows that the InVO_4_ electrode has excellent cycling stability, making it the perfect electrode for long‐term supercapacitors. These stated results of InVO_4_ electrode are due to admirable electrochemical property. Moreover, InVO_4_ showed high Coulombic efficiency.^[^ ^53^, ^54^ ^]^ This is based on the galvanostatic charge/discharge curves which were calculated to be 89% and 85% for InVO_4_ and InVO_4_–SiO_2_, respectively (Figure 9b).

**Figure 10:**
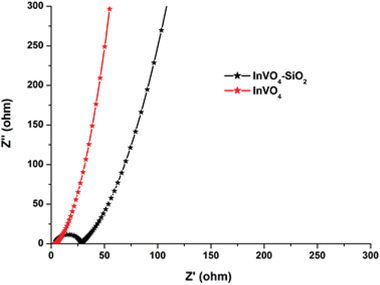
Impedance spectra of InVO_4_ and InVO_4_–SiO_2._

Measurements of electrochemical impedance spectroscopy (EIS) can be useful in exploring electrode electrical conductivity and electrode ion transmission performances. **Figure** **10** displays the Nyquist impedance plots calculated at an open‐circuit voltage of ≈5 mV in the regular three‐electrode configuration within a frequency region between 100 kHz and 10 MHz. The impedance spectrum can be divided into three parts with the so‐called knee frequency, the higher frequency semicircle arc, and the low‐frequency straight line. The intercept in the real part (*Z*′′) is the combination of three parts, such as i) electrolyte ionic strength resistance, ii) intrinsic resistance of the substrate (electrode materials), and iii) contact resistance between the active modified electrode material and the assisted current collector in the high‐frequency range. With the increase of InVO_4_ microsphere, the contact resistance of InVO_4_ composites grows progressively. An intercept on the real high‐frequency axis of the semicircle reflects the equivalent series resistance (*R*_s_), and the semicircle diameter represents the electrode's charge‐transfer resistance (*R*_ct_). The semicircle arc identified at the high‐frequency region results in a parallel combination of the charge‐transfer resistance (*R*_ct_) induced by Faradaic reactions. The charge‐transfer resistance (*R*_ct_) eventually decreases as the InVO_4_ content increases, also the conductivity of composite film improves. The *R*_ct_ value is negligible because of InVO_4_'s excellent electron mobility. At low frequencies, it appears as a vertical line, demonstrating an excellent capacitive output with no diffusion constraints.

## 3 Conclusions

In summary, InVO_4_ microsphere structures were successfully prepared with a simple surface–interface hydrothermal method, which is essential to achieve the hierarchical architectures. A specific capacity of 1710 F g^−1^ at 5 mV S^−1^ is resulted for InVO_4_ hybrid nanostructure with the improved electrochemical efficiency and enhanced cyclic stability (95% capacitor retention for 4000 cycles) compared to InVO_4_–SiO_2_. The improved electrochemical efficiency of InVO_4_ electrodes was attributed to their intrinsic pseudocapacitive properties. The results show that InVO_4_ and InVO_4_–SiO_2_ microspheres can be used for high‐performance supercapacitor applications. This research can provide experimental insights into the design of InVO_4_‐based nanocomposites, which shows the sufficient electrochemical capacitance for supercapacitors with the encapsulation of the SiO_2_ network.

## 4 Experimental Section

##### Materials

All the chemicals purchased were analytically classified and used without further purification. Double distilled water was used for all the experiments. Indium(III) chloride tetrahydrate, ammonium metavanadate, and 1*H*,1*H*,2*H*,2*H*‐*per*‐fluorooctyltriethoxysilane were obtained from Aldrich Chemicals and used as received. Sodium hydroxide and ethanol were purchased from Aldrich chemicals.

##### Preparation of InVO_4_ Hierarchical Microstructures

The representative fabrication procedure was as follows: 1 mmol of InCl_3_·4H_2_O was dissolved in 50 mL of deionized water, and a certain quantity of NH_4_VO_3_ (1 mmol) was dissolved in 50 mL of 5% HNO_3_. After the complete dissolution, the NH_4_VO_3_ solution was added dropwise to the InCl_3_·4H_2_O solution under vigorous stirring, and the resulting suspension pH was adjusted to 6.5 with 1.0 m aqueous NaOH. The mixture was stirred for 4 h to get the yellowish suspension. The resulting solution was transferred to a Teflon‐lined stainless‐steel autoclave (100 mL) and kept in an oven at 200 °C for 24 h, and then cooled down to room temperature. InVO_4_ precipitate was collected together with the excess of unreacted VO^4−^ and In^3+^ ions, and washed several times with deionized water and absolute ethanol before drying for 4 h in air at 80 °C. The sample was calcinated for 12 h at 500 °C to get InVO_4_ hierarchical microspheres and microrods.

##### Characterizations

Various analytical techniques were used to characterize the as‐prepared InVO_4_. SEM (Hitachi S‐4100) was used to analyze the surface morphology of the material. Prior to SEM measurements, samples were mounted on a platform of carbon film, followed by platinum coating for 5 min with a magnetron sputter. TEM (JEM‐2100, JEOL, Japan) was used to study the detailed morphological and diffractions of InVO_4_ hierarchical structures. X‐ray powder diffraction patterns were recorded using a Rigaku D/max‐2500 X‐ray diffractometer (Japan) with a Ni‐filtered Cu Kα radiation of (λ) 1.54178 Å. Fourier‐transform infrared spectroscopy/Attenuated total reflection (Thermo Nicolet 6700 FTIR), UV–visible diffuse reflectance spectroscopy (DRS) (TU−1901, Pgener al), and X‐ray photoelectron spectra (ESCA Lab 220i‐XL, VG Scientific XPS), were collected. For XPS, MgKα X‐ray was an excitation source with a base pressure of ≈3 × 10^−9^ mbar. Atomic force microscopy (AFM) analysis was performed by Bruker Corporation of Germany using a DI Multimode 8 scan microscope to measure the surface roughness of the materials.

## Conflict of Interest

The authors declare no conflict of interest.

## Supporting information

Supporting Information
