# Supplementary material for: Fabrication of Hierarchical Indium Vanadate Materials for Supercapacitor Application
Source: Glob Chall. 2020 Sep 28;4(11):2000002. doi: 10.1002/gch2.202000002 (PMC7607248; doi:10.1002/gch2.202000002)
Supplement: Supplementary file 1 — Supporting Information [file GCH2-4-2000002-s001.pdf]

© 2020 Wiley-VCH GmbH

Global Challenges
Open Access

Supporting Information

for *Global Challenges,* DOI: 10.1002/gch2.202000002

Fabrication of Hierarchical Indium Vanadate Materials for Supercapacitor Application

Balachandran Subramanian, Manimuthu Veerappan, Karthikeyan Rajan, Zheming Chen, Chengzhi Hu, Fei Wang, Feng Wang,* and Mingshu Yang**

Supporting Information

Fabrication of Hierarchical Indium vanadate materials for supercapacitor Application

Subramanian Balachandran,[a, b] Veerappan Manimuthu,[c] Karthikeyan Rajan, [d] Zheming Chen,[a] Chengzhi Hu,[b]* Wang Fei,[c] Feng Wang[a]*, Mingshu Yang [a]*

[a] Beijing National Laboratory for Molecular Sciences, Key Laboratory of Engineering Plastics, Institute of Chemistry, Chinese Academy of Sciences, Zhongguancun North First Street 2, Beijing 100190, P. R. China.

[b] Department of Mechanical and Energy Engineering, Southern University of Science and Technology, Nanshan District, Shenzhen, Guangdong, China

[c] Department of Electrical and Electronic Engineering, Southern University of Science and Technology, Nanshan District, Shenzhen, Guangdong, China

[d] Engineering Research Center for Hydrogen Energy Materials and Devices, College of Rare Earths (CORE), Jiangxi University of Science and Technology, Ganzhou 341 000, Jiangxi, China

*Corresponding author. Tel./fax: +86-10-62561945.

E-mail address: yms@iccas.ac.cn (**Mingshu Yang***), wangfeng0822@iccas.ac.cn (**Wang Feng***), hucz@sustc.edu.cn (**Chengzhi Hu***)

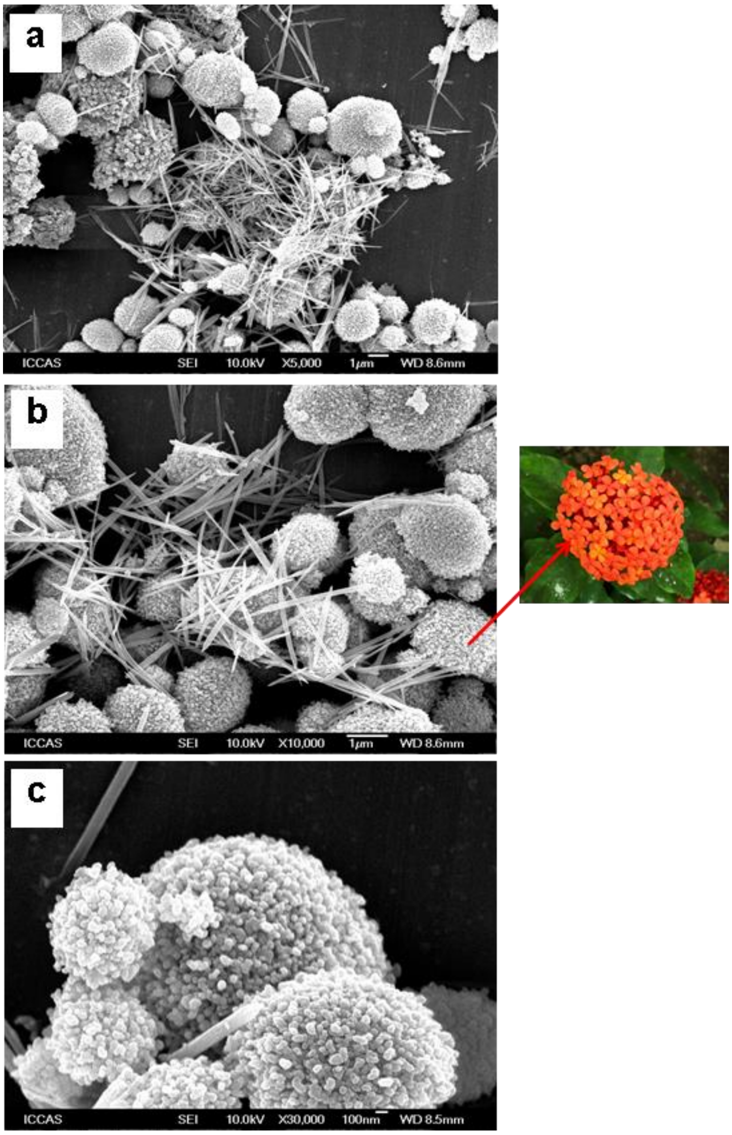

Figure S1. FESEM images of $InVO_4$ at different places (a-c) 10K

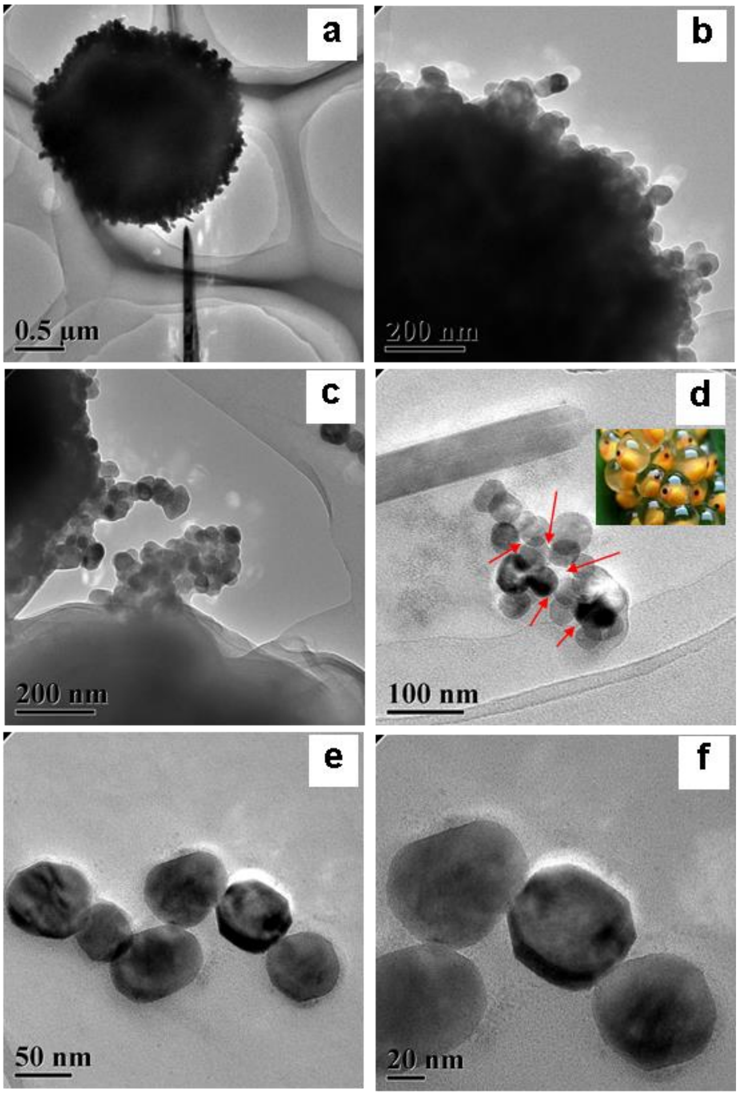

Figure S2. TEM images of $InVO_4$ at different magnifications (a) 0.5 µm, (b, c) 200 nm, (d) 100 nm, (e) 50 nm and (f) 20 nm.

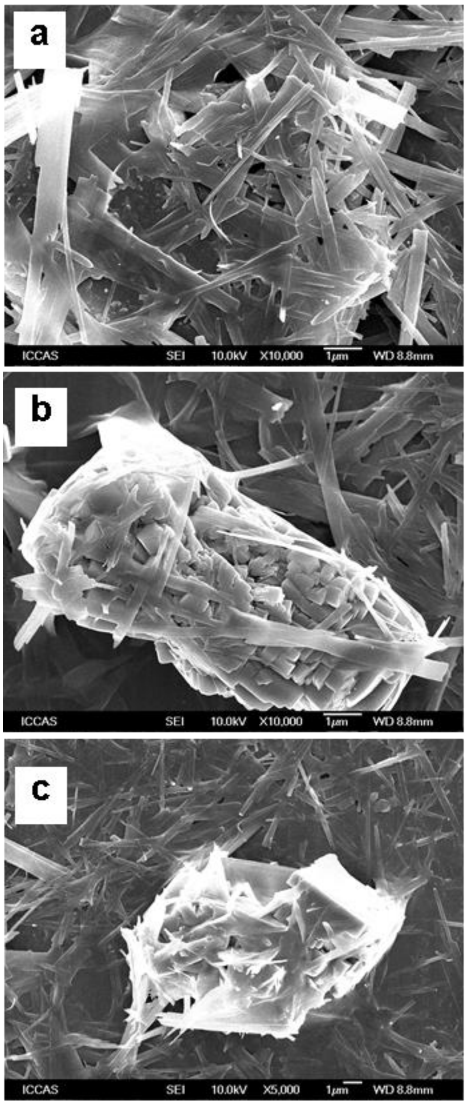

Figure S3. FESEM images of $InVO_4$-SiO_2 at different magnifications (a, b) 10K, and (c) 5 K.

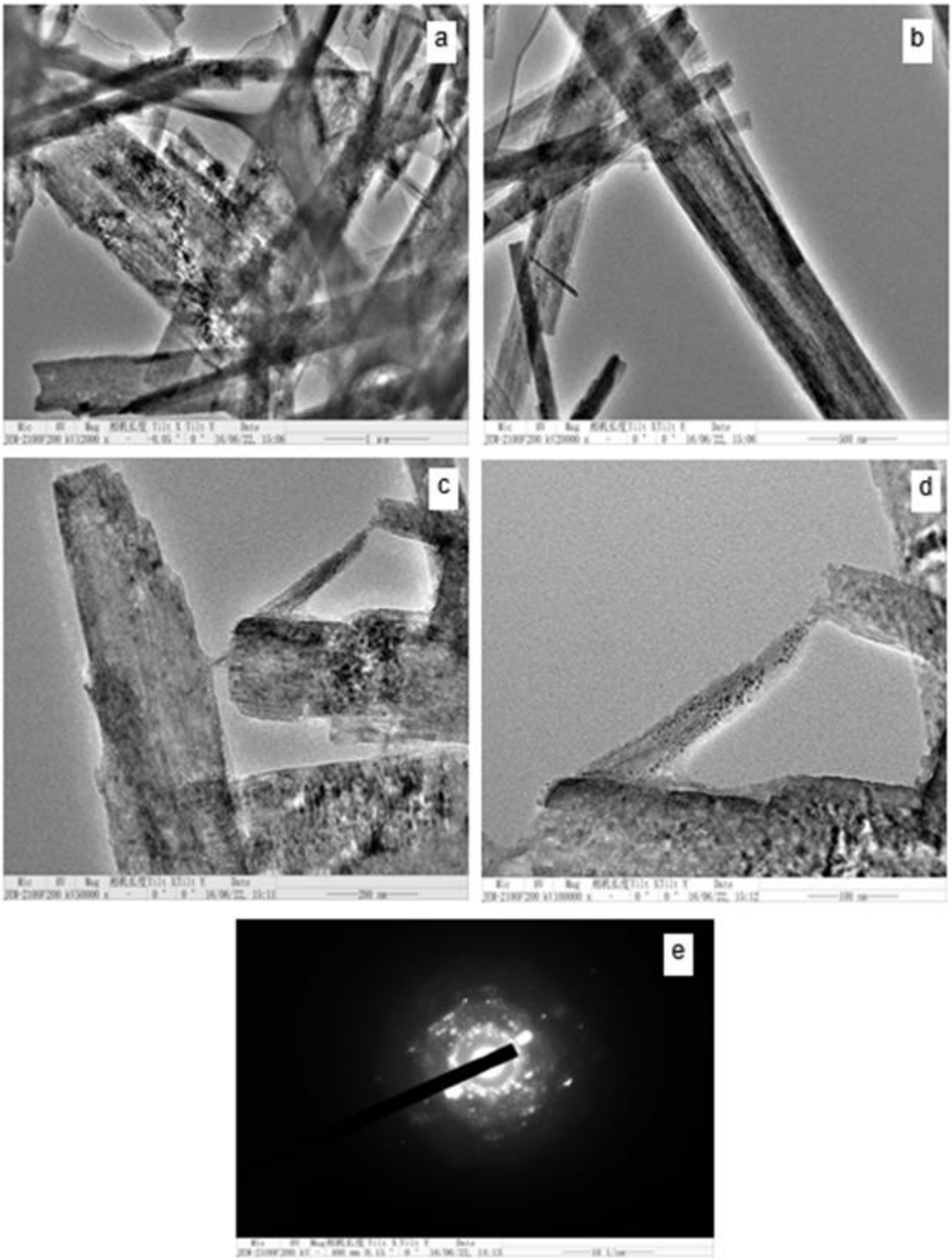

Figure S4. HR-TEM images of Silica encapsulated $InVO_4$ at different magnifications (a) 12 K, (b) 20 K, (c) 50 K (d) 100 K and (f) SAED pattern.

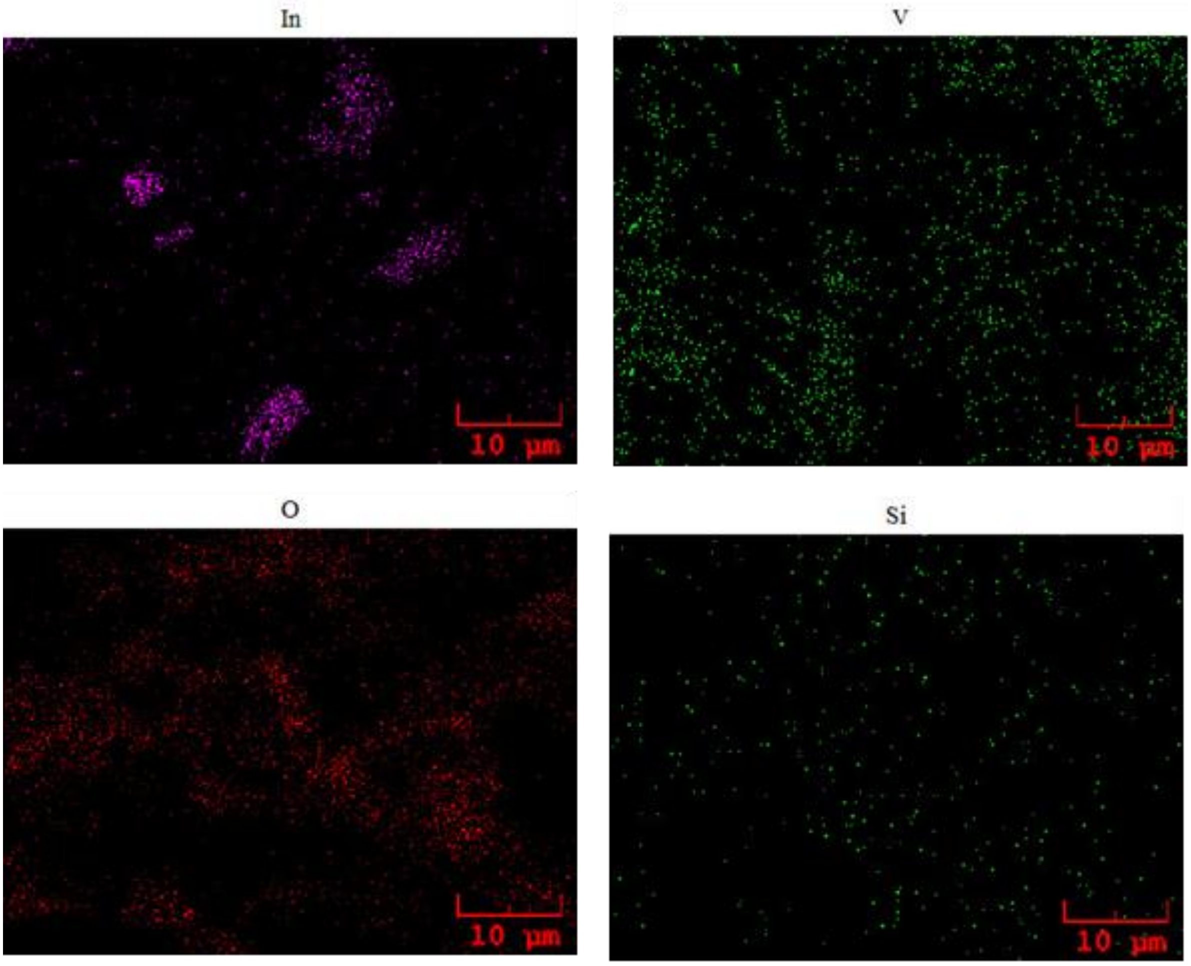

Figure S5. Elemental color mapping $InVO_4$ -SiO_2 from FESEM analysis

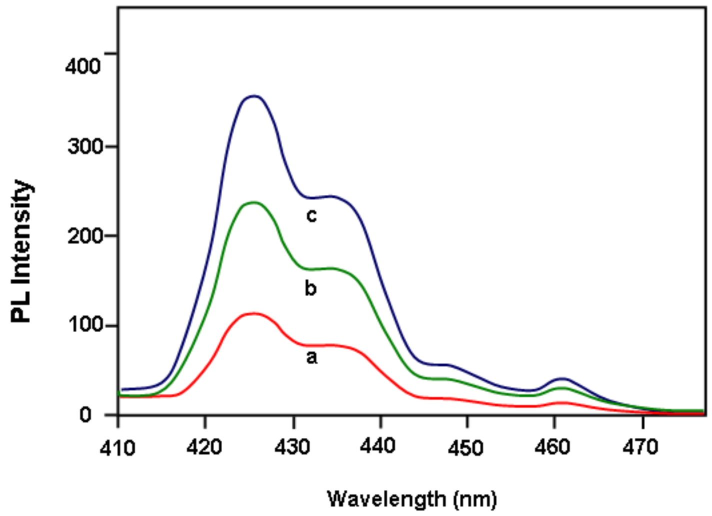

Figure S6. Photoluminescence spectra of (a) $InVO_4$, (b) commercial $InVO_4$ and (c) $InVO_4$- SiO_2 ,

Name	Start BE	Peak BE	End BE	Height CPS	FWHM eV	Area (P) CPS.eV	Area (N) TPP-2M	Atomic %
**C1s**	292.92	284.88	280.02	14628.41	1.58	30496.42	0.38	**30.35**
**In3d5**	448.67	444.95	442.5	3620.89	1.44	5874.47	0.06	**5.67**
**V2p**	527.77	517.14	511.57	45430.05	1.44	121235.72	0.18	**14.07**
**O1s**	536.77	529.88	526.87	64515.09	1.46	129241.45	0.62	**48.55**
**Si2p**	107.23	103.97	100.98	539.16	2.2	1294.82	0.02	**1.36**

Table 1: Elemental composition from XPS
